# The World Health Organization’s Global Monitoring System on Alcohol and Health

**DOI:** 10.35946/arcr.v35.2.15

**Published:** 2014

**Authors:** Vladimir Poznyak, Alexandra Fleischmann, Dag Rekve, Margaret Rylett, Jürgen Rehm, Gerhard Gmel

**Affiliations:** **Vladimir Poznyak, MD., Ph.D.,** *is the coordinator of Management of Substance Abuse unit at the World Health Organization, Geneva, Switzerland;*; **Alexandra Fleischmann, Ph.D.,** *is a technical officer at the Management of Substance Abuse unit at the World Health Organization, Geneva, Switzerland;*; **Dag Rekve, M.S.C.,** *is a technical officer at the Management of Substance Abuse unit at the World Health Organization, Geneva, Switzerland;*; **Margaret Rylett, M.A.,** *is a researcher at the Social and Epidemiological Research (SER) Department, Centre for Addiction and Mental Health (CAMH), Toronto, Canada;*; **Jürgen Rehm, Ph.D.,** *is director of the Social and Epidemiological Research Department at the Centre for Addiction and Mental Health, chair and professor in the Dalla Lana School of Public Health, University of Toronto, Canada, and section head at the Institute for Clinical Psychology and Psychotherapy, Technische Universität Dresden, Dresden, Germany.*; **Gerhard Gmel, Ph.D.,** *is a senior scientist at the Swiss Institute for the Prevention of Alcohol and Drug Problems, Lausanne, Switzerland.*

With growing awareness of the impact of alcohol consumption on global health (Rehm et al. 2004; World Health Organization [WHO] 2002, 2009) the demand for global information on alcohol consumption and alcohol-attributable and alcohol-related harm as well as related policy responses has increased significantly. Public health problems attributable to harmful alcohol consumption have become the focus of several World Health Assembly resolutions, including one adopted in 2005 that requested the Director-General of the WHO “to strengthen global and regional information systems through further collection and analysis of data on alcohol consumption and its health and social consequences, providing technical support to Member States and promoting research where such data are not available” (WHO 2005). Monitoring and surveillance are crucial in setting objectives for national alcohol plans and in evaluating success (for more details see Rehm and Scafato 2011). In recognition of the increasing demand from WHO Member States for global health information, the WHO’s 11th General Programme of Work called for monitoring health situations and assessing trends as one of six core functions for the period 2006–2015 (WHO 2006).

In 2010, the World Health Assembly endorsed the Global Strategy to Reduce the Harmful Use of Alcohol (WHO 2010), which targeted the monitoring and surveillance of harmful alcohol consumption and alcohol-attributable harm as one of 10 areas for action. The Global Strategy also identified production and dissemination of knowledge as one of the key components for global action (WHO 2010).

Most recently, the Political Declaration of the High-level Meeting of the United Nations General Assembly on the Prevention and Control of Non-Communicable Diseases (NCDs) mandated the development of a global monitoring framework, including indicators, and a set of voluntary global targets for the prevention and control of NCDs. This mandate explicitly mentioned the harmful use of alcohol as one of the four common risk factors for NCDs along with tobacco use, unhealthy diet, and lack of physical activity (United Nations 2011). This work yielded a set of nine voluntary targets, including at least a 10 percent relative reduction in the harmful use of alcohol and a set of 25 indicators, including the following possible indicators for monitoring the harmful use of alcohol as appropriate, within the national context: (1) total (recorded and unrecorded) alcohol per capita consumption (among those ages 15 and older) within a calendar year in liters of pure alcohol; (2) age-standardized prevalence of heavy episodic drinking among adolescents and adults; and (3) alcohol-related morbidity and mortality among adolescents and adults (WHO 2012). Inclusion of the alcohol target and indicators in the global monitoring framework for NCDs and their risk factors will increase the demand for high-quality global data on alcohol consumption and alcohol-related harm and attention to the WHO monitoring activities in this area.

## History of the WHO Global Monitoring System on Alcohol and Health

The WHO Program on Substance Abuse established the Global Alcohol Database in 1996, creating the world’s largest single source of information on levels and patterns of alcohol consumption, its health consequences, and policy responses in WHO Member States. Prior to this, WHO monitoring of alcohol and health activities largely was focused on collecting countries’ alcohol policy and prevention program data (Moser 1974, 1980, 1992). With the establishment of the Global Alcohol Database, the WHO Secretariat started to implement regular global questionnaire surveys on alcohol and health among the governmental officials of WHO Member States nominated to provide information to WHO in the areas of alcohol consumption, alcohol-related harm, and policy responses. The data collection tools were developed by WHO staff in collaboration with external experts. The first *Global Status Report on Alcohol* was published in 1999 (WHO 1999), followed by the *Global Status Report on Alcohol and Young People* (WHO 2001). In 2004, the WHO produced two global status reports based on the data collected from Member States and other sources during 2002: one on alcohol consumption and related harm (WHO 2004*a*) and the second focused on alcohol policy (WHO 2004*b*). The latest *Global Status Report On Alcohol and Health* contained newly developed country profiles (see figure) based on 30 key indicators related to alcohol consumption, health consequences, and policy responses (WHO 2011). The reports also provided valuable information on levels and patterns of alcohol consumption at global and regional levels, and contained estimates of alcohol-attributable disease burden. In 2006, the WHO Expert Committee on Problems Related to Alcohol Consumption recommended the establishment of a global information system on alcohol, based on the current WHO Global Alcohol Database, to continue efforts to collect, compile, and analyze alcohol monitoring and surveillance information based on comparable data and agreed definitions (WHO 2007).

## WHO Global Information System on Alcohol and Health

The WHO created the Global Information System on Alcohol and Health (GISAH) to collect, compile, analyze, and disseminate global information on alcohol and health. From the very beginning of its development by the WHO Department of Mental Health and Substance Abuse in collaboration with the Centre for Addiction and Mental Health (CAMH) in Canada, the global information system was conceived as integrated with the regional information systems on alcohol, although at that time such a system existed only in the WHO European region. GISAH now is part of the WHO Global Health Observatory and integrates four regional information systems from countries in the Americas, Europe, Southeast Asia, and Western Pacific regions (http://www.who.int/gho/alcohol/en/index.html). The GISAH functions as one single data repository, with common data collection and data quality–control procedures to prevent discrepancies between the global and regional information systems on alcohol and health.

Within GISAH, data are organized under a broad set of seven categories of indicators: levels of alcohol consumption; patterns of consumption; harms and consequences; economic aspects; alcohol control policies; prevention, research, and treatment resources; and youth and alcohol.

GISAH currently encompasses more than 150 alcohol-related indicators, with data for more than 225 countries and territories and includes indicators that are comparable across countries. The information on prevention and treatment resources is presented in another information system (i.e., Resources for the Prevention and Treatment of Substance Use Disorders) (http://www.who.int/gho/substance_abuse/en/index.html), which also is a part of the WHO Global Health Observatory.

Since its development, the GISAH and its regional components have become the central global information tool for dynamic presentation of worldwide data on levels and patterns of alcohol consumption, alcohol-attributable health and social consequences, and policy responses at all levels. The WHO Global Strategy to reduce the harmful use of alcohol explicitly mentions strengthening the GISAH and developing or refining appropriate data-collection mechanisms, based on comparable data and agreed indicators and definitions, as the key activity of the WHO Secretariat in support of WHO Member States in producing and disseminating knowledge on alcohol and health (WHO 2010).

Among the remaining key challenges for improving international comparisons of data on alcohol consumption and alcohol-attributable health consequences are the following: (1) national monitoring systems on alcohol and health in many countries are weak, fragmented or lacking; (2) difficulties exist in estimating consumption of informally and illicitly produced alcohol; (3) poor comparability of indicators used in different jurisdictions; (4) limited geographical representation of studies on the association of alcohol consumption with health outcomes; and (5) a paucity of international multi-country research projects on alcohol epidemiology using common research protocols.

## Processes and Procedures Underlying the WHO GISAH

Data sources for the GISAH include results of the WHO Global Survey on Alcohol and Health; government documents and national statistics available in the public domain; data from the Global Burden of Disease Project; data from national and international surveys including questions on alcohol consumption and related harm from the WHO STEPS (http://www.who.int/chp/steps/instrument/en/index.html) survey instrument; and data in the public domain from economic operators in alcohol production and trade, including industry-supported organizations, published scientific articles, data from the United Nations (UN) Food and Agricultural Organization (FAO) and other UN agencies, and intergovernmental organizations such as Organization International de la Vigne et du Vin. The Canadian CAMH conducts passive surveillance of the relevant published as well as grey literature. The WHO Secretariat convenes regular meetings with key data providers on alcohol consumption to discuss and triangulate available data for achieving better estimates when national data are either unavailable or incomplete.

The WHO Global Survey on Alcohol and Health, a key data-collection mechanism, is implemented in collaboration with WHO regional and country offices, the Canadian CAMH and several other academic centers and institutions. The WHO Global Survey Instrument on Alcohol and Health, developed by WHO in collaboration with all partners involved in the survey, is forwarded to all WHO Member States through the WHO regional and country offices for completion by focal points and national counterparts explicitly nominated by governments to collaborate with WHO on this activity. For countries belonging to the European Union (EU), the survey is implemented in collaboration with and support from the European Commission. In 2008, the survey instrument contained 69 questions grouped into three sections: (1) alcohol policy; (2) alcohol consumption; and (3) alcohol-related health indicators. The questionnaire was developed in English and translated into French, Portuguese, Russian, and Spanish. In 2008, completed questionnaires were received from 84 percent of WHO Member States, representing 97 percent of the world’s population. In 2012, 177 Member States participated in the survey, which represented a 90 percent response rate and covered 98 percent of the world population.

In 2012, the survey tool was modified to strengthen the alcohol policy section in line with the main suggested areas for national action specified in the WHO Global Strategy to reduce the harmful use of alcohol. In 2012 the survey was partially implemented using the Web-based data-collection tool.

### Alcohol Per Capita Consumption

One of the most important indicators of alcohol consumption in the Global Survey on Alcohol and Health is per capita consumption (among those aged 15 and older) in liters of pure alcohol. Notwithstanding some limitations associated with its aggregate-level nature (Bloomfield et al. 2003), alcohol per capita consumption is a key indicator for measuring levels of alcohol exposure in populations (WHO 2000, 2007). Despite the potential measurement bias in unrecorded consumption, per capita consumption is considered the most reliable and valid indicator for alcohol consumption in a population (Gmel and Rehm 2004) and is particularly appropriate for monitoring purposes. Population-based survey data are extremely important for further estimates of alcohol consumption in different age and gender groups but currently cannot be considered as a valid and reliable basis for estimates of alcohol per capita consumption at country, regional, and global levels. Surveys are thought to underestimate per capita consumption by more than 50 percent (Midanik 1988, 1982; Rehm et al. 2007) and survey errors are larger (Shield and Rehm 2012).

The alcohol per capita consumption indicator is based on the estimates of per capita consumption of recorded and unrecorded alcohol, the latter referring to alcohol that is not taxed and is outside the usual system of governmental control, because it is produced, distributed, and sold outside formal channels and, therefore, not registered by routine data collection (Rehm et al. 2003, 2007). It is critical to include unrecorded consumption in the estimates of overall levels of alcohol exposure in populations, because more than one-fourth of global consumption stems from unrecorded alcohol (WHO 2011). However, contrary to some conjectures, unrecorded consumption does not seem to be linked to more health problems than recorded consumption, if volume and patterns of drinking are controlled for (Rehm et al. 2010). Recorded consumption can be measured via sales and taxation or via production, export, and import. Many national governments regularly monitor alcohol per capita consumption, and reliable data is available from a significant number of countries, though predominantly high-income. These national statistics, if based on validated methodology, are given highest preference in reporting in GISAH. However, even if data on alcohol consumption are unavailable from national statistics, *per capita* consumption can be estimated, either via industry data in the public domain, or by using data supplied from by the FAO and its statistical database (FAOSTAT) (http://faostat3.fao.org/home/index.html). An algorithm is used by the WHO Secretariat to decide which statistics to give preference to, depending on the validity of the data (see http://who.int/gho/gisah).

Unrecorded consumption obviously is harder to estimate and monitor at the country level. Only a few countries have regular monitoring of unrecorded consumption. For all others, unrecorded alcohol consumption is estimated based on one-time studies and expert opinion. For the 2012 Global Survey on Alcohol and Health an additional questionnaire component on unrecorded alcohol consumption has been developed and implemented based on the principles of the Delphi survey methodology (for a description, see Linstone and Turoff 1975; Rehm and Gadenne 1990). The questionnaire in this component covers estimates of unrecorded alcohol consumption in its major categories, such as home production (of spirits, wine, and beer), alcohol brought over the border (smuggling, duty free, and cross-border shopping), illegal production (including counterfeit alcoholic beverages), and surrogate alcohol (liquids usually containing ethanol and industrial spirits not intended for consumption as beverages). The questionnaire also addresses perceived importance of unrecorded alcohol consumption from a public health perspective as well as the measures implemented at the country level to reduce the public health impact of illicit and informally produced alcohol in line with a set of policy options and interventions listed in the Global Strategy to reduce the harmful use of alcohol (WHO 2010).

Tourist consumption also is being considered in estimating alcohol per capita consumption in populations, where tourist consumption is significant (because the number of tourists per year is at least the number of inhabitants) and is not balanced by drinking by national inhabitants abroad during vacations. This mainly is the case for smaller countries.

Alcohol per capita consumption is one example of the approximately 200 indicators monitored via the GISAH at the country, regional, and global levels.

### Data Validation

Before releasing the national, regional, and global data on alcohol consumption, alcohol-related harm, and policy responses, the WHO Secretariat undertakes an intensive process of data validation by compiling country profiles with all the available data for key indicators and forwarding the country profiles to each country for validation. At this stage, any discrepancies are resolved by considering new data for the periods covered in the survey, further triangulation of available information, and building consensus around disputed qualitative indicators. After the validation process, the data are uploaded in the WHO GISAH and subsequently presented in the WHO global status reports on alcohol and health.

## Further Developments

Both the depth of the GISAH and the rigor of data collection and validation make it an indispensable tool for policy development and evaluation, as well as for global research (e.g., the Global Burden of Disease 2010 estimates were based on WHO global monitoring [Lim et al. 2012; Shield et al. 2013]). One of the key tasks for the WHO in the area of global monitoring and surveillance on alcohol and health is to support the development of effective national systems for monitoring alcohol consumption, its health and social consequences and related policy responses, while also strengthening national capacity for analyzing and disseminating the information, also through the WHO’s global and regional information systems on alcohol and health. To further improve comparability of data generated in countries, consistent data collection mechanisms, agreed indicators and definitions, and enhanced dissemination of data is needed. The new alcohol module in the WHO STEPS instrument (http://www.who.int/chp/steps/instrument/en/index.html), which is the main data collection tool used in WHO surveillance activities on risk factors for chronic diseases, is an attempt to prioritize some well-defined key indicators and improve consistency between the relevant national surveillance activities and the WHO global monitoring system on alcohol and health. Work continues on WHO tools for alcohol epidemiology and monitoring, such as the *International Guide for Monitoring Alcohol Consumption and Related Harm* (WHO 2000).

One of the challenges for the WHO global monitoring system on alcohol and health continues to be a time lag between the alcohol exposure data collected from countries and their dissemination through GISAH and WHO global and regional status reports on alcohol and health. Efforts to reduce this time lag will involve data collection through Web-based data collection tools, optimizing data validation and dissemination procedures, as well as strengthening partnerships and resource mobilization for effective functioning of the global monitoring system.

The ultimate objective for the WHO global monitoring system on alcohol and health is strengthening the link between monitoring activities and policy development and evaluation. This system, which includes the global surveys, GISAH, and WHO global status reports on alcohol and health, is the central mechanism for monitoring implementation of the WHO global strategy to reduce the harmful use of alcohol and report on its implementation to WHO Member States (WHO 2013), other constituencies, and the public health community at large.

## Figures and Tables

**Figure:**
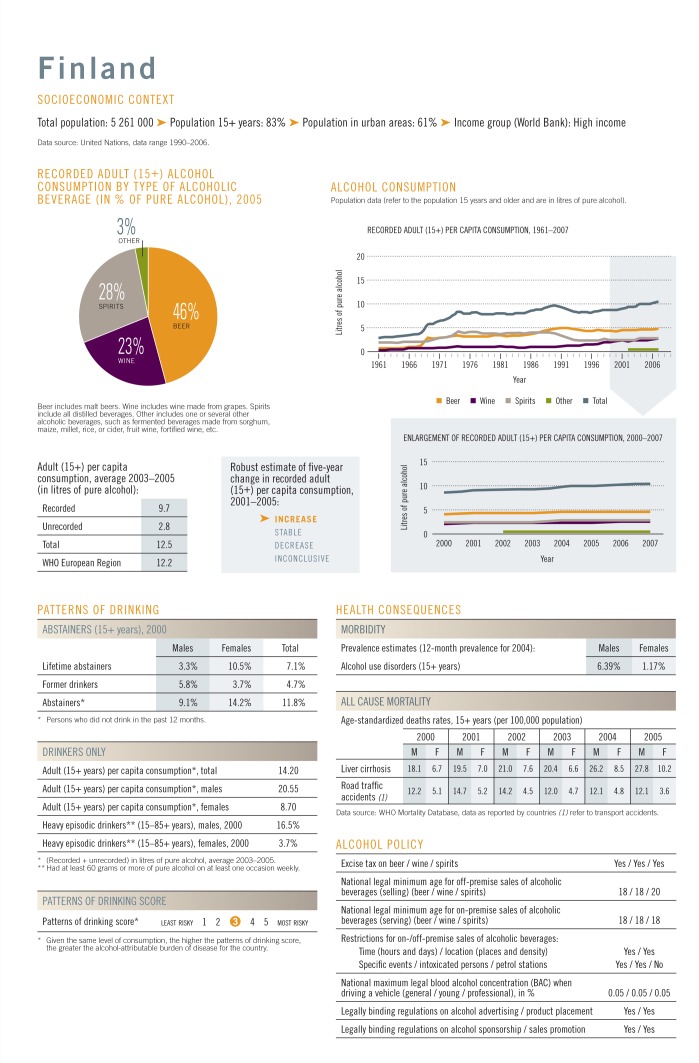
Example of country profile as presented in the *WHO Global Status Report on Alcohol and Health* (WHO 2011) (reproduced with permission from the WHO).
